# Primary adrenal mature teratoma: A rare case report and review of literature

**DOI:** 10.1016/j.amsu.2022.103422

**Published:** 2022-03-01

**Authors:** Imane Assarrar, Marouane Harhar, Dounia Zerrouki, Najat Draoui, Achraf Miry, Amal Bennani, Siham Rouf, Tijani El Harroudi, Hanane Latrech

**Affiliations:** a Department of Endocrinology-Diabetology and Nutrition, Mohammed VI University Hospital Center, Oujda, Morocco; b Faculty of Medicine and Pharmacy, Mohammed First University, Oujda, Morocco; c Department of Surgical Oncology, Regional Oncology Center, Mohammed VI University Hospital Center, Oujda, Morocco; d Laboratory of Anatomic Pathology, Mohammed VI University Hospital Center, Oujda, Morocco; e Laboratory of Epidemiology, Clinical Research and Public Health, Faculty of Medicine and Pharmacy, Mohammed First University, Oujda, Morocco

**Keywords:** Case report, Teratoma, Retroperitoneal tumor, Adrenal gland tumor, Adrenalectomy, LC-MS/MS, liquid chromatography with tandem mass spectrometry, CLIA, Chemiluminescence immunoassay, CMIA, chemiluminescent microparticle immunoassay, VMA, vanillyl mandelic acid

## Abstract

**Introduction:**

Teratoma is a germ cell tumor, deriving from totipotent cells. Teratomas usually occur in gonads and are rarely extra-gonadal. The adrenal location is exceptional. Only few cases of primary adrenal teratomas have been reported in literature, mainly in young patients.

**Case report:**

We report the case of a 56-year-old female patient who presented with pyrosis, dyspepsia and abdominal pain that was evolving for 5 months. The abdominal computed tomography revealed a voluminous mass of the right adrenal gland and the hormonal evaluation was normal. The patient underwent an open transperitoneal adrenalectomy and the histopathological examination of the specimen confirmed the diagnosis of mature teratoma.

**Discussion:**

Adrenal teratomas are commonly asymptomatic and their diagnosis depends mostly on radiologic findings. Malignant transformation is very rare. Surgical excision is the mainstay of treatment with a good prognosis.

**Conclusion:**

Open surgery should always be considered in large and adhering teratoma tumors of the adrenal gland. Some pathologic features and tumorigenesis of adrenal teratomas are not entirely elucidated, thus the importance of larger studies in order to comprehend this pathological entity.

## Highlights

• The adrenal location of primary adrenal teratomas is exceptional.
• The preoperative diagnosis is challenging, based mainly on imaging findings.
• Open surgery should always be considered in large and adhering teratoma tumors of the adrenal gland.

## 1 Introduction

Teratomas are germ cell tumors deriving from totipotent cells. In adults, these tumors occur regularly in gonads and are rarely extra-gonadal. Retroperitoneal teratomas account for 4% of all primary teratomas and are more common in children, whereas adrenal teratomas are even rarer [1]. Based on the degree of their differentiated tissue, they are classified as mature or immature. Mature teratomas are made of at least 90% of well-differentiated tissues, while immature teratomas comprise at least 10% of immature tissue and are at greater risk for malignant transformation [2]. The preoperative diagnosis is challenging as these tumors can be often mistaken for other adrenal tumors such as adrenal adenomas, myelolipomas or angiomyolipomas [3].

In this paper, we report the case of a mature adrenal teratoma in an adult female patient with a systematic literature review. This case has been reported following the SCARE criteria [4].

## 2 Case report

**Fig. 1:**
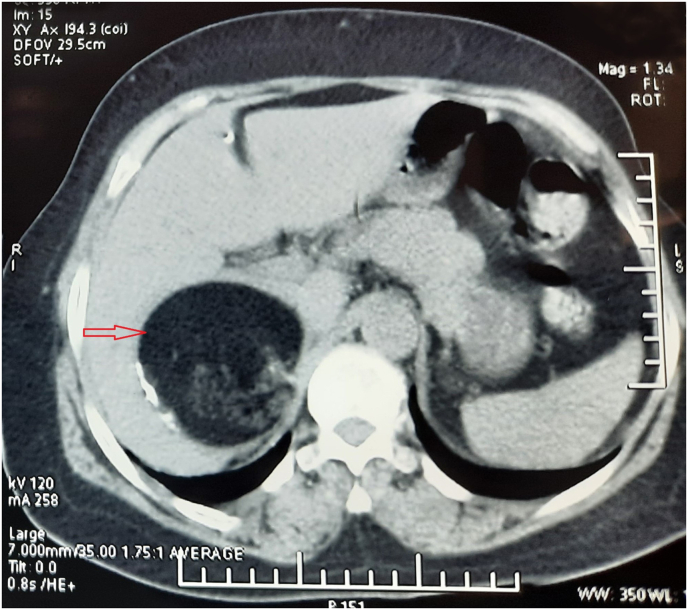
Axial image of abdominal CT scan showing a mass of the right adrenal gland (red arrow) with major fatty component and a minimal soft tissue density, including a macrocalcification. (For interpretation of the references to colour in this figure legend, the reader is referred to the Web version of this article.)

A 56-year-old female patient with unremarkable personal and family history presented with right side upper abdominal pain that appeared 5 months before. The physical examination was normal. The abdominal contrast-enhanced computed tomography (CT) revealed a large spherical mass of the right adrenal gland, consisting majorly of fatty component, with minimal soft tissue density and a large calcification, sized 76 × 72 × 80 mm (depth x width x height) (Fig. 1). The tumor was responsible for liver scalloping in the segments I, VI, VII and VIII and was intimately attached to the posterior border of the inferior vena cava (IVC).

Routine biological assessment, including liver and kidney function tests and complete blood count, was unremarkable. Adrenal-related hormonal assessment was normal: 24-h urine metanephrine was at 0.27 μmol/24h (NV: 0.20–1.00 μmol/24h, LC-MS/MS) and normetanephrine at 0.31 μmol/24h (NV: 0.40–2.10 μmol/24h, LC-MS/MS). In the supine position, aldosterone was at 169 pmol/l (NV: 83–405 pmol/l, CLIA), renin was at 4.5 mUI/l (NV: 2.8–39.9 mU/l, direct immunometric assay) with an aldosterone-to-renin ratio at 34 (NV < 64). In the upright position, aldosterone was at 461 pmol/l (NV: 208–1000 pmol/l, CLIA), renin was at 39.7 mUI/l (NV: 4.4–46.1 mUI/l, direct immunometric assay) with an aldosterone-to-renin ratio at 12 (NV < 64). Moreover, 24-h urinary free cortisol was normal at 630 ng/24h (43–1760 ng/24h, immunoassay), and morning serum cortisol was at 14ng/ml after 1 mg dexamethasone suppression test (NV < 18 ng/ml, CMIA). These results excluded a functional adrenal tumor. Tumor markers were normal: alpha-fetoprotein (AFP) was at 1.5 ng/ml (NV: 0–7 ng/ml, CMIA), human chorionic gonadotropin (hCG) was at 1.99 mUI/ml (NV < 10 mUI/ml, CMIA), CA19-9 was at 5.32 U/ml (NV: 0–33 U/ml, CMIA) and carcinoembryonic antigen (CEA) was at 1.94 ng/ml (NV < 5 ng/ml, CMIA).

**Fig. 2:**
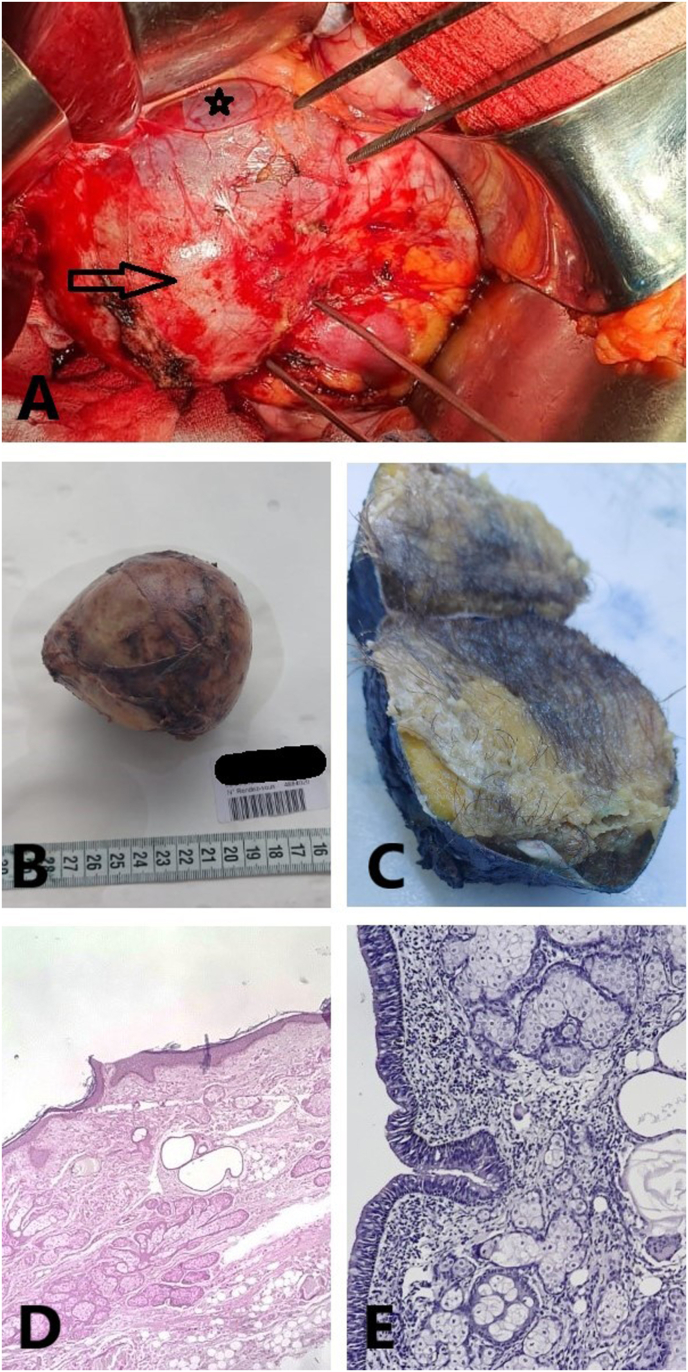
A. Intraoperative image showing the adrenal mass (arrow) intimately adhering to the IVC (star). B. Macroscopic image of the specimen showing a gray well-encapsulated mass. C. Dissection of the mass showing the presence of hair, sebaceous material and bone fragments. D. Microphotography showing presence of a normal squamous epithelium with presence of adnexal elements: sebaceous and sweat glands. HE; 100X. E. Microphotography showing the focal presence of a respiratory epithelium. The lamina propria contains sebaceous glands. HE; 200X.

Thus, a transperitoneal adrenalectomy under general anesthesia, in the left lateral decubitus position, was performed by the surgical oncology department. Intraoperatively, the tumor was difficult to mobilize as it was intimately adherent to the IVC, the right liver and the right pillar of the diaphragm (Fig. 2). The mass was gently dissected and successfully removed. The postoperative course was uneventful.

The postoperative gross examination showed a gray well-encapsulated mass, measuring 8 × 6 × 7 cm and weighing 247g. Upon dissection, the tumor contained hair, sebaceous material and bone fragments. Microscopically, the tumor included sebaceous and sweat glands, respiratory epithelium, cartilage and bone material (Fig. 2). The diagnosis of multitissular mature teratoma was confirmed. Neither immature nor malignant components were identified. Postoperatively, the patient remained well and exhibited no signs of recurrence during 18 months of follow-up.

## 3 Discussion

Teratomas are germ cell tumors composed of mature tissues arising from more than one germinal layer i.e., ectoderm, mesoderm, and endoderm. Teratomas occurring in early childhood are mostly extra-gonadal, while those occurring in adults are mainly found in the gonads [1]. The retroperitoneal location, particularly in the adrenal gland, is extremely rare accounting for 1–11% of all retroperitoneal primary tumors [2].

For our systematic review of primary adrenal teratomas in adults, we searched for the terms of “adrenal teratoma” in the PubMed database. A total of 289 studies were published between June 1952 and December 2021. We excluded the cases reported in children, review articles, letters, abstracts without full text and irrelevant publications. Out of the remaining studies, we identified 40 cases of primary adrenal teratomas in adults aged more than 16 years reported in 19 publications. We extracted and summarized demographic, clinical, imaging, management and follow-up data in Table 1.

**Table 1:** Characteristics of published cases of primary adrenal teratomas in adults.

Case No	Author/Year	Age	Gender	Clinical symptoms	Maximal diameter (mm)	Laterality	Solid vs cystic	Histologic Diagnosis	Calcifications	Surgery	Follow-up (months)
1 [5]	Lam et al., 1999	18	F	GP	110	L	S	–	Yes	–	7
2 [5]	Lam et al., 1999	17	M	GP	75	R	C	MT	–	–	6
3 [5]	Lam et al., 1999	37	F	GP	100	L	C	MT	–	–	8
4 [6]	Bedri et al., 2002	57	F	AP	80	L	C	MT	Yes	–	–
5 [7]	Polo et al., 2004	21	F	AP	380	L	C	MT	No	OA	–
6 [8]	Castillo et al., 2006	61	F	IF	80	L	S	MT	–	LA	–
7 [9]	Rais-Bahrami et al., 2007	33	M	IF	50	L	C S	MT	Yes	OA	–
8 [10]	Sato et al., 2010	37	M	IF	200	L	C S	MT	Yes	LA	–
9 [11]	Giordano et al., 2011	62	M	IF	47	L	C S	MT	Yes	LA	–
10 [12]	Bhatti et al., 2013	22	M	FP	108	L	C	MT	Yes	–	–
11 [13]	Li H et al., 2015	49	M	IF	110	R	C	MT	Yes	LA	12
12 [14]	Li S et al., 2015	21	F	–	85	R	C S	MT	Yes	LA	–
13 [14]	Li S et al., 2015	16	F	–	90	R	C S	MT	Yes	LA	–
14 [14]	Li S et al., 2015	43	F	–	49	L	S	MT	No	LA	–
15 [14]	Li S et al., 2015	49	F	–	53	L	C S	MT	Yes	LA	–
16 [14]	Li S et al., 2015	51	F	–	24	R	–	–	–	LA	–
17 [15]	Nadeem et al., 2015	19	M	FP	80	R	C S	MT	Yes	OA	–
18 [16]	Bhatia et al., 2016	24	F	FP	76	L	S	MT	Yes	OA	29
19 [17]	Kataoka et al., 2016	32	F	IF	51	R	C S	MT	Yes	LA	8
20 [18]	Kuo et al., 2017	26	M	FP	85	R	–	MT	Yes	OA	–
21 [18]	Kuo et al., 2017	29	F	IF	25	L	–	MT	Yes	LA	–
22 [18]	Kuo et al., 2017	24	F	AP	115	L	C	MT	–	LA	–
23 [19]	Niu et al., 2017	36	F	IF	80	R	S	MT with adenocarcinoma	Yes	LA	–
24 [20]	Pandit et al., 2018	16	F	AL	120	L	C	MT	Yes	LA	–
25 [21]	Ramakant et al., 2018	25	F	FP	190	R	S C	MT	Yes	OA	12
26 [22]	Zhou et al., 2018	69	F	IF	100	L	S	MT	Yes	OA	12
27 [22]	Zhou et al., 2018	29	F	IF	25	L	–	MT	Yes	LA	–
28 [23]	Ban et al., 2019	60	M	FP FP	120 58	L L	C S C S	MT	Yes Yes	LA	–
29 [24]	Wang et al., 2019	22	F	IF	132 125	L R	C S	MT	Yes	LA	–
30 [25]	Zhong et al., 2020	59	F	FP	106	L	C	MT	Yes	OA	124
31 [25]	Zhong et al., 2020	54	F	AS	102	R	S C	MT	Yes	OA	124
32 [25]	Zhong et al., 2020	22	F	AP	100	R	S C	MT	Yes	OA	109
33 [25]	Zhong et al., 2020	48	F	AS	96	R	C	MT	Yes	LA	101
34 [25]	Zhong et al., 2020	26	M	FP	40	R	C	MT	Yes	LA	90
35 [25]	Zhong et al., 2020	18	F	AP	88	R	S C	MT	Yes	LA	88
36 [25]	Zhong et al., 2020	55	F	AS	140	L	S	MT	Yes	OA	69
37 [25]	Zhong et al., 2020	28	F	AS	45	R	S C	MT	Yes	LA	60
38 [25]	Zhong et al., 2020	29	F	AS	78	R	C	MT	Yes	LA	56
39 [25]	Zhong et al., 2020	29	F	AS	70	L	S C	MT	Yes	LA	56
40 [25]	Zhong et al., 2020	72	F	AS	60	R	S	MT	No	OA	55
41 [25]	Zhong et al., 2020	28	F	AS	90,5	L	C	MT	Yes	OA	7
42 [25]	Zhong et al., 2020	41	F	AS	180	L	C	MT	Yes	OA	43
43 [25]	Zhong et al., 2020	45	M	AS	60,8	L	C	MT	Yes	LA	28
44 [3]	Cheng He et al., 2020	17	F	AS	70	R	C	MT	Yes	LA	12
45 [26]	Tojal et al., 2020	75	M	AS	50,6	L	C	MT	Yes	LA	20
46 Our case	Assarrar et al., 2021	56	F	FP	80	R	S	MT	Yes	OA	18

AP, abdominal pain; IF, incidental finding; FP, flank pain; EP, epigastric pain; AL, abdominal lump; AD, abdominal distention; –, not recorded; L, left; R, right; S, solid; C, cystic; MT, mature teratoma; AS Asymptomatic; OA, open adrenalectomy; LA, laparoscopic adrenalectomy.

According to literature, primary adrenal teratomas in adults are extremely rare with only 41 cases reported cases in the 20th century, in patients aged between 16 and 75 years. They can occur in a wide age range, with a peak in the third decade of life and a mean age of 33.2 years, and seem to affect more females than males with a male-female sex-ratio of 0.31 (11 males and 35 females). As regards laterality, 52.2% of the masses occurred in the left adrenal gland, while 46.3% were found in the right side including our case. One case presented with bilateral adrenal teratomas [24].

In terms of clinical presentation, primary adrenal teratomas can be discovered fortuitously (22 cases), or can present with non-specific symptoms like abdominal pain when the diameters become large enough to exert pressure on neighboring organs (19 cases). The average tumor size was 6.23cm. The largest reported mass was sized 38 cm and weighed 10kg [16].

Adrenal teratomas are typically non-functional tumors. In the available data, thirty patients, including our case, have benefited from a preoperative endocrine evaluation that was strictly normal, except one case of a 36-yar-old patient who had abnormal laboratory values of cortisol, adenocroticotropic hormone (ACTH), aldosterone, and vanillyl mandelic acid (VMA) due to a malignant transformation of a mature adrenal teratoma with adenocarcinoma components [19]. Tumor markers, AFP and hCG were tested in 14 patients, with negative results.

Abdominal CT is the gold standard for preoperative diagnosis. However, depending on the composition of the mass, the diagnosis is usually difficult. Classically, these tumors present as well-circumscribed masses with mixed density secondary to the presence of multiple tissue types (i.e., calcification, bone, teeth, soft tissue and fluid components) and a predominant fatty density [27]. Davidson et al. [28] suggested that the presence of a heterogeneous mass containing a well-circumscribed fluid volume, fat-fluid level (created by adipose tissue or sebaceous material) and calcifications were the most characteristic radiologic findings on CT. The presence of major fatty component can mislead the diagnosis as myelolipomas have similar radiologic features. The calcifications can be linear, radial or punctuate. They were found in 33 cases (71.2%), while fat component was identified in 38 cases (82.6%), including our case. Magnetic resonance imaging confers a better identification of soft tissues and has superior tumor staging assessment [27]. Abdominal ultrasound has a small contribution to the diagnosis as it can only differentiate between cystic and solid elements [26].

Our patient underwent open surgery for her large adhering mass. Currently, laparoscopic surgery has become the mainstay of treatment for benign adrenal tumors dethroning open surgery, since it has lower morbidity and allows an earlier recovery with less pain [25]. However, open surgery should be the first choice for large tumors with important adherence, such as the tumor described in our case. In the available literature, all patients underwent surgical excision of the tumor. Depending on the size, 18 patients (39.1%) had open surgery and 28 patients (60.9%) went through laparoscopic surgery (Table 1).

Histopathological examination is the key for conclusive diagnosis of adrenal teratoma. Mature teratomas appear as well-encapsulated tumors, with smooth appearance, mainly cystic and composed by two or three germ layers, usually comprising fully mature elements i.e., sebaceous material, hair, bone and fat components. Immature teratomas include at least 10% of undifferentiated tissue [14,29].

Gatcombe et al. [2] reported that the malignancy rate of retroperitoneal teratomas in adults was significantly higher than in children (26% vs. 7%). In our review data, the diagnosis of mature adrenal teratoma was confirmed in all patients except two cases; a case of a 17-year-old patient who had a malignant teratoma that needed postoperative chemotherapy and a 36-year-old female patient whose diagnosis was a mature adrenal teratoma with adenocarcinoma [19,30]. The criteria for benign teratomas are: the absence of malignant or immature elements in histopathology, the absence of metastasis, normal levels of AFP and hCG and the absence of recurrences during follow-up [13].

The overall prognosis after complete surgical excision of adrenal teratomas is favorable. However, whether the teratoma is mature or immature, a regular post-operative follow-up is mandatory for early identification and management of recurrences and metastases [14]. The follow-up of our patient for 18 months didn't reveal any recurrence or metastasis.

## 4 Conclusion

Teratomas rarely present as retroperitoneal tumors in adults, particularly in adrenal glands. Their preoperative diagnosis remains difficult and its confirmation depends solely on histopathological examination. Surgical management of mature adrenal teratomas is the mainstay of treatment with an excellent postoperative prognosis. Several clinical and pathologic features of adrenal teratomas are not clearly elucidated. Further studies are necessary in order to comprehend their tumorigenesis and pathologic properties.

## Sources of funding

This research was not funded.

## Consent

A written informed consent was obtained from the patient for publication of this case report and accompanying images. A copy of the written consent is available for review by the Editor-in-Chief of this journal on request.

## Ethical approval

This is a case report that does not require a formal ethical committee approval. Data were anonymously registered in our database. Access to data was approved by the head of the department.

## Author contribution

Dr. Imane Assarrar wrote the manuscript.

Dr. Marouane Harhar helped in writing and provided surgical data.

Dr. Dounia Zerrouki helped in writing and literature review.

Dr. Najat Draoui helped in writing and literature review.

Dr. Achraf Miry interpreted and provided pathological data.

Pr. Amal Bennani confirmed the histological diagnosis and revised the final manuscript.

Pr. Siham Rouf helped in writing, supervised the redaction and revised the manuscript.

Pr. Tijani El Harroudi operated the patient and revised the final manuscript.

Pr. Hanane Latrech helped in writing, supervised the redaction, revised and approved the final draft for publication.

All authors approved the final version of the manuscript.

## Registration of research studies

This is not an interventional study. We only reported the patient's findings from our database as a case report.

## Guarantor

Professor Hanane Latrech.

## Provenance and peer review

Not commissioned, externally peer reviewed.

## Declaration of competing interest

The authors declare no conflicts of interest.
